# Molecular epidemiology of methicillin-resistant Staphylococcus aureus.

**DOI:** 10.3201/eid0702.010236

**Published:** 2001

**Authors:** B Shopsin, B N Kreiswirth

**Affiliations:** Public Health Research Institute and Department of Microbiology, New York University School of Medicine, New York, New York, USA.

## Abstract

Subtyping methicillin- resistant Staphylococcus aureus (MRSA) isolates and tracking nosocomial infections have evolved from phenotypic to genotypic approaches; most laboratories now depend on pulsed-field gel electrophoresis (PFGE). We discuss the limitations of current image-based genotyping methods, including PFGE, and the advantages (including ease of entering data into a database) of using DNA sequence analysis to control MRSA infections in health-care facilities.


### Molecular Epidemiology of Methicillin-Resistant *Staphylococcus aureus*

**Bo Shopsin*† and Barry N. Kreiswirth***
*Public Health Research Institute and †Department of Microbiology,
New York University School of Medicine, New York, New York, USA

Subtyping methicillin-resistant *Staphylococcus aureus* (MRSA) isolates and tracking nosocomial infections have evolved from phenotypic to genotypic approaches; most laboratories now depend on pulsed-field gel electrophoresis (PFGE). We discuss the limitations of current image-based genotyping methods, including PFGE, and the advantages (including ease of entering data into a database) of using DNA sequence analysis to control MRSA infections in health-care facilities.

*Staphylococcus aureus* is a major nosocomial pathogen that causes a range of diseases, including endocarditis, osteomyelitis, pneumonia, toxic-shock syndrome, food poisoning, carbuncles, and boils. In the early 1950s, acquisition and spread of beta-lactamase-producing plasmids thwarted the effectiveness of penicillin for treating *S. aureus* infections. In 1959, methicillin, a synthetic penicillin, was introduced. However, by 1960, methicillin-resistant *S. aureus* strains were identified, the direct result of *S. aureus* acquiring the *mecA* gene, which encodes for an altered penicillin-binding protein gene (PBP2a) (1).

By the early 1960s, European hospitals were reporting outbreaks of MRSA infections, and subsequently MRSA clones spread to health-care institutions around the world (2). In the United States, MRSA is responsible for approximately 25% of nosocomial infections, and reports of community-acquired MRSA infections are increasing (3). The multidrug-resistant phenotype of MRSA strains and their intrinsic beta-lactam resistance make them difficult and costly to treat (4,5). In some medical institutions in New York City, MRSA accounts for approximately 29% of nosocomial infections and 50% of associated deaths (5). Controlling MRSA remains a primary focus of most hospital infection control programs (6).

Bacterial strain typing, or subspeciation, has become an important clinical tool to investigate suspected outbreaks and to evaluate nosocomial transmission. Numerous typing methods focus on discriminating MRSA isolates. We discuss the limitations of current image-based genotyping methods and the advantages of using DNA sequence analysis to control MRSA infections in health-care settings.

### Genotyping Aims

Bacterial strain typing distinguishes epidemiologically related or clonal isolates from unrelated isolates. Epidemiologically related isolates are viewed as descendants from a common precursor cell; thus, their genomic "fingerprints" will be indistinguishable but recognizably different from unrelated or random isolates from the same species (7). Outbreak investigations of *S. aureus* and other nosocomial pathogens are viewed as short-term events or cases of local epidemiology, and in these settings most genotyping methods are able to distinguish clonal spread from unrelated isolates.

Understanding genetic relatedness becomes more challenging when the strain study population is larger, separated further in time, and recovered from a larger geographic area. The long-term or global epidemiologic question is whether the strains causing disease in one geographic area are related to those causing disease in other regions. A combination of genotyping methods has been used to study global *S. aureus* transmission (8,9).

In addition to tracking outbreaks, genotyping is used to distinguish between contaminating and infecting isolates and between separate episodes of infection and relapse of disease (10). Genotyping is also able to link certain *S. aureus* clonal types and disease syndromes, such as in cases of food poisoning and toxic-shock syndrome. The present challenge is to continue to build bacterial databases linking genetic markers and clinical presentations so that important correlates of disease can be identified.

### Typing *Staphylococcus aureus*

Numerous techniques are available to differentiate *S. aureus*, and specifically MRSA, isolates. Historically, isolates were distinguished by phenotypic methods, including antibiotic susceptibility testing and bacteriophage typing. Both methods have limitations, as genetically unrelated isolates commonly have the same antibiogram, and many *S. aureus* isolates are nontypeable by phage typing (7).

With the advent of molecular biology, strain typing focused on DNA-based methods. Initial techniques compared restriction endonuclease patterns of chromosomal or plasmid DNA. The second generation of genotyping methods included Southern blot hybridization using gene-specific probes, ribotyping, polymerase chain reaction (PCR)-based approaches, and pulsed-field gel electrophoresis (PFGE) (10,11). These methods require subjective interpretation and comparison of patterns and fingerprint images. The ability to digitize and store images and to compare patterns by using matching software programs has enhanced these methods. However, they still remain difficult to standardize between laboratories, and the image-based information is difficult to

Address for correspondence: B. N. Kreiswirth, Public Health Research Institute Tuberculosis Center, 455 First Ave., New York, NY 10016, USA; fax: 212-578-0853; e-mail: barry@phri.org organize for rapid search and retrieval by computer. In addition, image-based methods do not provide biological criteria to evaluate the relatedness between different strains (12).

Binary typing is a more objective genotyping method that compares the presence or absence of 12 different targets in the *S. aureus* genome. The binary coding system for each probe creates a numerical type amenable to relational databases (13). However, binary typing fails to provide information on genetic relatedness between strain types. This method is also technically subjective, as it requires interpretation of a positive hybridization signal from background.

### DNA Sequence Analysis

DNA sequence analysis is an objective genotyping method; the genetic code (A-T-C-G) is highly portable and easily stored and analyzed in a relational database. Recent advances in DNA sequencing technology, including rapid, affordable, high-throughput systems, have made it possible for sequencing to be considered as a viable typing method.

Sequencing the same DNA targets from disparate isolates and then cataloging mutation patterns constitute an approach termed comparative sequencing. Two different strategies have been used to provide genotyping data: multilocus sequence typing (MLST), which compares sequence variation in numerous housekeeping gene targets, and single-locus sequence typing, which compares sequence variation of a single target.

MLST has been developed for *Neisseria gonorrhoeae*, *Streptococcus pneumoniae,* and *S. aureus,* based on the classic multilocus enzyme electrophoresis (MLEE) method used to study the genetic variability of a species. Sequence analysis of five to seven housekeeping genes provides a database from which to infer relationships in somewhat distantly related isolates that have had substantial time to diversify (14,15).

The MLST approach is too labor-intensive, time-consuming, and costly to use in a clinical setting. More than 2,500 bp must be compared for each isolate. In addition, for certain recent subpopulations, such as MRSA, genetic variability in the housekeeping targets will likely be limited and discrimination will be restricted. However, a single-locus target, if discriminating, provides an inexpensive, rapid, objective, and portable genotyping method to subspeciate bacteria. Using a single target depends on finding a region for sequencing that is sufficiently polymorphic to provide useful strain resolution. Loci with short sequence repeat (SSR) regions may have suitable variability for discriminating outbreaks (16). Two *S. aureus* genes conserved within the species, protein A (*spa*) and coagulase (*coa*), have variable SSR regions constructed from closely related 24- and 81-bp tandem repeat units, respectively. In both genes, the in-frame SSR units are degenerative, variable in number, and variable in the order in which repeat units are organized. The genetic alterations in SSR regions include both point mutations and intragenic recombination that arise by slipped-strand mispairing during chromosomal replication and that result in a high degree of polymorphism (17,18).

Frenay et al. (19) compared the SSR region in the protein A gene in a collection of MRSA isolates studied by classical bacteriophage typing and showed that *spa*-typing clustered isolates previously grouped to phage type III-29. However, van Belkum et al. questioned whether the repeat region was too hypervariable and thus not a sound target for epidemiologic studies (20).

### Validation of *spa*-Typing

The *spa*-type database of the Public Health Research Institute Tuberculosis Center includes >950 clinical *S. aureus* isolates; most (~80%) are methicillin resistant. DNA sequence analysis identified 37 unique, 24-bp SSR types; one type was 1 codon longer. The number and organization of the repeat types define the *S. aureus spa* type; to date, 186 *spa* types have been identified and catalogued in a relational database.

To further evaluate the clinical and epidemiologic validity of the protein A repeat region as a genotyping tool, we analyzed two historic collections previously characterized by multiple genetic methods. The Centers for Disease Control and Prevention's (CDC) collection of 59 staphylococci included 58 *S. aureus* isolates, 37 of them methicillin resistant; 29 isolates had been previously grouped into four identifiable clusters based on sound epidemiologic links. These isolates have been repeatedly studied with an array of techniques (e.g., phage typing, antibiograms, PFGE, ribotyping; Table 1). In the CDC collection, 22 SSR regions and 13 different *spa* types were identified. *spa*-typing correctly classified 27 of the 29 outbreak cultures and incorrectly grouped four isolates to these clusters. Overall, *spa*-typing produced results better than the mean score of 25 correct classifications and 5 misclassifications (18). These results support the findings of Frenay et al. (19) that *spa*-typing has the stability to correctly group epidemiologically related strains.

The second collection we analyzed consisted of 261 MRSA isolates from 12 New York City hospitals historically catalogued by Southern blot hybridization using *mecA* and Tn*554* and by PFGE. Together, these two methods identified 39 genotypes, which were further categorized into 96 PFGE subtypes. Five major MRSA clonal types were identified, and the predominant *mecA*:Tn*554*:PFGE genotype (I:A:A) was found at each of the 12 hospitals. Of the 261 MRSA isolates, 107 were typed as I:A:A. Twenty-two similar PFGE patterns were grouped to the "A" type (12). We named this genotype pattern, which has been routinely identified as the predominant MRSA clone in the United States, the "North American" MRSA clone (unpub. data). *spa*-typing correctly identified the five major MRSA clonal groups and also

Table 1. Comparison of typing methods used to discriminate *Staphylococcus aureus* strains

| Method | Total no. of types | No. classified | No. misclassified |
|---|---|---|---|
| Phage typing | 18 | 25 | 4 |
| Antibiogram | 21 | 26 | 6 |
| Biotype | 23 | 17 | 2 |
| Plasmids | 20 | 23 | 0 |
| *Hin*dIII ribotyping | 16 | 27 | 7 |
| *ClaI* ribotyping | 9 | 29 | 7 |
| IS typing | 9 | 16 | 3 |
| RFLP typing | 17 | 28 | 3 |
| *coa*-PCR | 7 | 28 | 8 |
| PFGE | 25 | 28 | 7 |
| FIGE[a] | 25 | 27 | 3 |
| Immunoblotting | 23 | 28 | 6 |
| MLEE | 21 | 26 | 4 |
| Range | 7-25 | 16-28 | 0-8 |
| Average | 18 | 25 | 5 |
| *spa*-typing | 16 | 27 | 4 |

[a]FIGE = field-inversion gel electrophoresis.

categorized 98 of the 107 I:A:A MRSA isolates as *spa* type 2; the remaining 9 isolates were distributed in five different *spa* types, which were grouped by repeat composition and repeat organization (Table 2).

To test the genetic validity of *spa*-typing, we sequenced the SSR region of the coagulase gene. The slower "clock-speed" of the larger coagulase repeat region provided an independent genetic target to compare evolutionary relatedness with the results provided by *spa*-typing. Sequence analysis revealed a common *coa* type among seven of the nine *spa* types (Table 2), providing additional, independent evidence of genetic relatedness of the I:A:A MRSA isolates.

The PFGE fingerprint patterns in the North American MRSA clonal types listed in Table 2 were determined (Figure). The isolates with *spa* types 14, 25, and 28 (lanes 5, 9, and 6, respectively) were grouped to the North American clonal type on the basis of *spa* composition and organization and *coa* type. However, these isolates were initially distinguished by different *mecA*:Tn*554* genotypes and more diverse PFGE patterns.

### Conclusions

The finding that *spa*-typing could genotype the *S. aureus* isolates from two different collections in congruence with established procedures disproves the belief that this repeat region is too unstable for epidemiologic studies. While *spa*-typing does not have the resolving power of PFGE subtyping, it is fast, easy to use and interpret, and compatible for building relational databases. Most importantly, DNA sequence analysis of the protein A repeat region provides an unambiguous, portable dataset that simplifies information sharing between laboratories and facilitates creating a large-scale database for studying global and local epidemiology.

The MLST database of *S. aureus* should establish a sound genetic framework to describe the species (14). These data are portable and can be easily linked to a *spa* and *coa* database, a process that takes advantage of the objective nature of sequence information. The *spa*-short repeat region and its variation in both composition and organization have established a library of distinguishable patterns that allows isolates to be easily and accurately genotyped. These groupings are supported by other image-based genotyping methods and sequencing secondary targets such as the coagulase short sequence repeats (17).

Although still limited in number, isolates that have been genotyped on the basis of *mecA*:Tn*554*:PFGE fingerprint

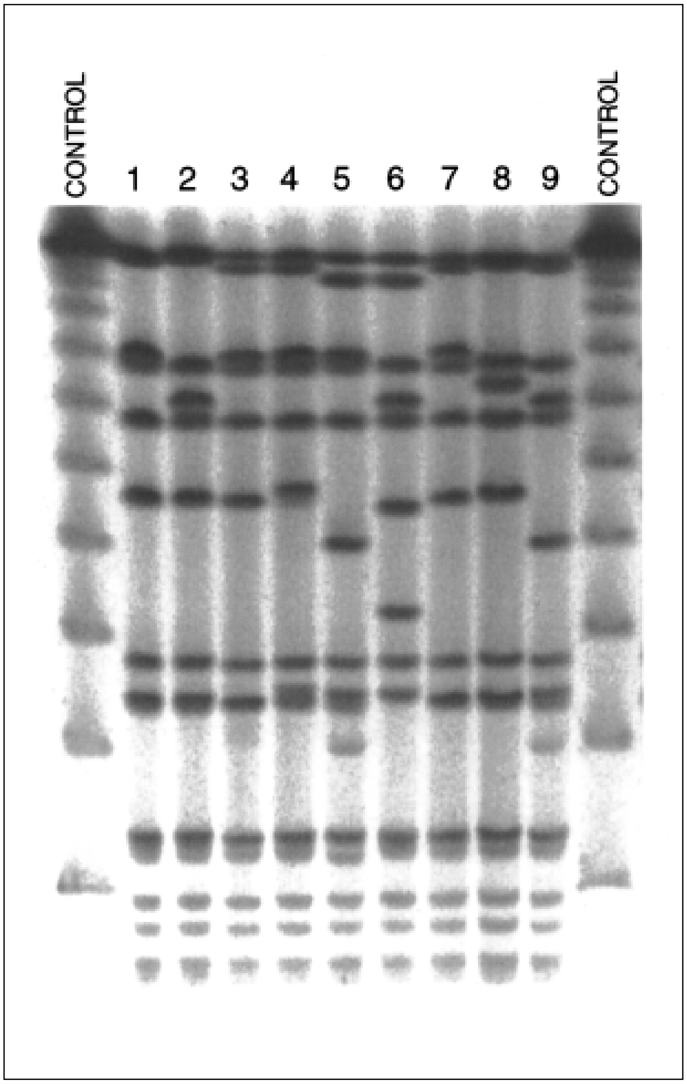

Figure. *Sma*I-pulsed-field gel electrophoresis of nine *Staphylococcus aureus* strains representative of the most prevalent MRSA clonal type in North America.

Table 2. Genotype of the "North American" MRSA clone

| Isolates (no. = 107) | mecA | Tn*554* | PFGE | *spa* type | *spa*-type repeats | *coa*-type repeats |
|---|---|---|---|---|---|---|
| 98 | I | A | A | *spa* type 2 | T-J-M-B-M-D-M-G-M-K | A-B-C-D-E-F |
| 2 | I | A | A | *spa* type 24 | T-J-M-E-M-D-M-G-M-K | |
| 1 | I | A | A | *spa* type 23 | T-J-M-B-M-D-M-G-K | A-B-C-D-E-F |
| 2 | I | A | A | *spa* type 29 | T-J-M-B-M-D-M-G-G-M-K | A-B-C-D-E-F |
| 1 | I | A | A | *spa* type 60 | T-J-M-A-M-G-M-K | |
| 3 | I | A | A | *spa* type 26 | T-J-M-B-M-G-M-K | A-B-C-D-E-F |
| New isolates | mecA | | PFGE | | | |
| 1 | II | NH | A' | *spa* type 14 | T-J-M-B-M-D-M-G-M-K-K | A-B-C-D-E-F |
| 1 | II | NH | A' | *spa* type 25 | T-J-M-D-M-G-M-K | A-B-C-D-E-F |
| 1 | III | NH | A' | *spa* type 28 | T-K-J-M-B-M-D-M-G-M-K-K | A-B-C-D-E-F |

patterns and *spa*- and coa-typing provide a rich database to study the recent spread of *S. aureus* and MRSA clones. These data provide sound evidence that *Sma*I-PFGE patterns change at a faster clock-speed than do the protein A SSRs and that the coagulase repeats change at a slower clock-speed than the shorter protein A repeats.

In short, sequence typing permits the widespread use of a proactive approach to investigate suspected outbreaks of MRSA.

Dr. Shopsin is completing his MD-PhD program at New York University Medical Center.

Dr. Kreiswirth is the founding director of the Public Health Research Institute Tuberculosis Center in New York City. The center was established in January 1992 in response to the reemergence of TB in New York. The focus of the laboratory centers on the molecular epidemiology of TB and nosocomial infections; research aims include identifying and developing genotyping targets to improve infection control and public health.

### References

1. Barber M. Methicillin-resistant Staphylococci. J Clin Pathol 1963;1:308-11.
2. Stewart GT, Holt RJ. Evolution of natural resistance to the newer penicillins. BMJ 1963;1:308-11.
3. Boyce JM, Jackson MM, Pugliese G, Batt MD, Fleming D, Garner JS, et al. Methicillin-resistant *Staphylococcus aureus* (MRSA): a briefing for acute care hospitals and nursing facilities. The AHA Technical Panel on Infections Within Hospitals [see comments]. Infect Control Hosp Epidemiol 1994;15:105-15.
4. Hacek DM, Suriano T, Noskin GA, Kruszynski J, Reisberg B, Peterson LR. Medical and economic benefit of a comprehensive infection control program that includes routine determination of microbial clonality. Am J Clin Pathol 1999;111:647-54.
5. Rubin RJ, Harrington CA, Poon A, Dietrich K, Greene JA, Moiduddin A. The economic impact of *Staphylococcus aureus* infection in New York City Hospitals. Emerg Infect Dis 1999;5:9-17.
6. Scheckler WE, Brimhall D, Buck AS, Farr BM, Friedman C, Garibaldi RA, et al. Requirements for infrastructure and essential activities of infection control and epidemiology in hospitals: a consensus panel report. Society for Healthcare Epidemiology of America [see comments]. Infect Control Hosp Epidemiol 1998;19:114-24.
7. Maslow JN, Mulligan ME, Arbeit RD. Molecular epidemiology: application of contemporary techniques to the typing of microorganisms [see comments]. Clin Infect Dis 1993;17:153-62.
8. Roman RS, Smith J, Walker M, Byrne S, Ramotar K, Dyck B, et al. Rapid geographic spread of a methicillin-resistant *Staphylococcus aureus* strain. Clin Infect Dis 1997;25:698-705.
9. Roberts RB, Tennenberg AM, Eisner W, Hargrave J, Drusin LM, Yurt R, et al. Outbreak in a New York City teaching hospital burn center caused by the Iberian epidemic clone of MRSA. Microb Drug Resist 1998;4:175-83.
10. Tenover FC, Arbeit RD, Goering RV. How to select and interpret molecular strain typing methods for epidemiological studies of bacterial infections: a review for healthcare epidemiologists. Molecular Typing Working Group of the Society for Healthcare Epidemiology of America. Infect Control Hosp Epidemiol 1997;18:426-39.
11. Kreiswirth B, Kornblum J, Arbeit RD, Eisner W, Maslow JN, McGeer A, et al. Evidence for a clonal origin of methicillin resistance in *Staphylococcus aureus*. Science 1993;259:227-30.
12. Roberts RB, de Lencastre A, Eisner W, Severina EP, Shopsin B, Kreiswirth BN, et al. Molecular epidemiology of methicillin-resistant *Staphylococcus aureus* in 12 New York hospitals. MRSA Collaborative Study Group. J Infect Dis 1998;178:164-71.
13. van Leeuwen W, van Belkum A, Kreiswirth B, Verbrugh H. Genetic diversification of methicillin-resistant *Staphylococcus aureus* as a function of prolonged geographic dissemination and as measured by binary typing and other genotyping methods. Res Microbiol 1998;149:497-507.
14. Enright MC, Day NP, Davies CE, Peacock SJ, Spratt BG. Multilocus sequence typing for characterization of methicillin-resistant and methicillin-susceptible clones of *Staphylococcus aureus*. J Clin Microbiol 2000;38:1008-15.
15. Maiden MC, Bygraves JA, Feil E, Morelli G, Russell JE, Urwin R, et al. Multilocus sequence typing: a portable approach to the identification of clones within populations of pathogenic microorganisms. Proc Natl Acad Sci U S A 1998;95:3140-5.
16. Van Belkum A, Scherer S, van Alphen L, Verbrugh H. Short-sequence DNA repeats in prokaryotic genomes. Microbiol Mol Biol Rev 1998;62:275-93.
17. Shopsin B, Gomez M, Waddington M, Riehman M, Kreiswirth BN. The use of coagulase gene (*coa*) repeat region nucleotide sequences for the typing of methicillin-resistant *Staphylococcus aureus*. J Clin Microbiol 2000;38:3453-56.
18. Shopsin B, Gomez M, Montgomery SO, Smith DH, Waddington M, Dodge DE, et al. Evaluation of protein A gene polymorphic region DNA sequencing for typing of *Staphylococcus aureus* strains. J Clin Microbiol 1999;37:3556-63.
19. Frenay HM, Bunschoten AE, Schouls LM, Van Leeuwen WJ, Vandenbroucke-Grauls CM, Verhoef J, et al. Molecular typing of methicillin-resistant *Staphylococcus aureus* on the basis of protein A gene polymorphism [see comments]. Eur J Clin Microbiol Infect Dis 1996;15:60-4.
20. van Belkum A, Riewerts Eriksen N, Sijmons M, Van Leeuwen W, Van den Bergh M, Kluytmans J, et al. Are variable repeats in the spa gene suitable targets for epidemiological studies of methicillin-resistant *Staphylococcus aureus* strains? [letter; comment]. Eur J Clin Microbiol Infect Dis 1996;15:768-70.
